# The human Stat1 gain-of-function T385M mutation causes expansion of activated T-follicular helper/T-helper 1-like CD4 T cells and sex-biased autoimmunity in specific pathogen-free mice

**DOI:** 10.3389/fimmu.2023.1183273

**Published:** 2023-05-19

**Authors:** Ori Scott, Shagana Visuvanathan, Emily Reddy, Deeqa Mahamed, Bin Gu, Chaim M. Roifman, Ronald D. Cohn, Cynthia J. Guidos, Evgueni A. Ivakine

**Affiliations:** ^1^ Division of Immunology and Allergy, Department of Paediatrics, Hospital for Sick Children and University of Toronto, Toronto, ON, Canada; ^2^ Program for Genetics & Genome Biology, Hospital for Sick Children Research Institute, Toronto, ON, Canada; ^3^ Institute of Medical Science, University of Toronto, Toronto, ON, Canada; ^4^ Program in Developmental and Stem Cell Biology, The Hospital for Sick Children, Toronto, ON, Canada; ^5^ Department of Obstetrics, Gynecology and Reproductive Biology, Michigan State University, East Lansing, MI, United States; ^6^ Institute for Quantitative Health Science and Engineering, Michigan State University, East Lansing, MI, United States; ^7^ The Canadian Centre for Primary Immunodeficiency and The Jeffrey Modell Research Laboratory for the diagnosis of Primary Immunodeficiency, The Hospital for Sick Children, Toronto, ON, Canada; ^8^ Division of Clinical & Metabolic Genetics, Department of Paediatrics, Hospital for Sick Children and University of Toronto, Toronto, ON, Canada; ^9^ Department of Immunology, University of Toronto, Toronto, ON, Canada; ^10^ Department of Physiology, University of Toronto, Toronto, ON, Canada

**Keywords:** STAT1, autoimmunity, chronic activation, T helper, immune dysregulation, Specific pathogen free (SPF), mouse model, gain of function (GOF)

## Abstract

**Introduction:**

Humans with gain-of-function (GOF) mutations in STAT1 (Signal Transducer and Activator of Transcription 1), a potent immune regulator, experience frequent infections. About one-third, especially those with DNA-binding domain (DBD) mutations such as T385M, also develop autoimmunity, sometimes accompanied by increases in T-helper 1 (Th1) and T-follicular helper (Tfh) CD4 effector T cells, resembling those that differentiate following infection-induced STAT1 signaling. However, environmental and molecular mechanisms contributing to autoimmunity in STAT1 GOF patients are not defined.

**Methods:**

We generated Stat1T385M/+ mutant mice to model the immune impacts of STAT1 DBD GOF under specific-pathogen free (SPF) conditions.

**Results:**

Stat1T385M/+ lymphocytes had more total Stat1 at baseline and also higher amounts of IFNg-induced pStat1. Young mutants exhibited expansion of Tfh-like cells, while older mutants developed autoimmunity accompanied by increased Tfh-like cells, B cell activation and germinal center (GC) formation. Mutant females exhibited these immune changes sooner and more robustly than males, identifying significant sex effects of Stat1T385M-induced immune dysregulation. Single cell RNA-Seq (scRNA-Seq) analysis revealed that Stat1T385M activated transcription of GC-associated programs in both B and T cells. However, it had the strongest transcriptional impact on T cells, promoting aberrant CD4 T cell activation and imparting both Tfh-like and Th1-like effector programs.

**Discussion:**

Collectively, these data demonstrate that in the absence of overt infection, Stat1T385M disrupted naïve CD4 T cell homeostasis and promoted expansion and differentiation of abnormal Tfh/Th1-like helper and GC-like B cells, eventually leading to sex-biased autoimmunity, suggesting a model for STAT1 GOF-induced immune dysregulation and autoimmune sequelae in humans.

## 1 Introduction

Humans with gain-of-function (GOF) mutations in *Signal Transducer and Activator of Transcription (STAT)1* exhibit widespread immune dysregulation. The major function of this STAT family transcription factor is to mediate the biological effects of pro-inflammatory cytokines, such as interferons (IFN), and to a lesser extent interleukin 6 (IL-6), which are produced when certain cells sense microbial products (1, 2). Although transcriptional complexes containing unphosphorylated STAT1 can mediate baseline expression of IFN-stimulated genes (ISG) (3–6), canonical infection-induced STAT1-mediated ISG expression involves a phosphorylation cascade following IFN receptor ligation. Type I IFNα/β cytokines activate Janus kinases (JAK) to phosphorylate STAT1 and STAT2, promoting their dimerization to induce transcription of genes that limit viral replication, enhance CD8 T cell cytotoxic effector function, and induce CD4 T cells to provide B cell help (7). IFNγ (Type II IFN) induces pSTAT1 homodimers to activate macrophage/monocytes and dendritic cell pro-inflammatory functions, enhance antigen presentation and secretion of cytokines that promote differentiation of Th1 cells from naïve CD4 T cells (8). IL6 activates STAT1 and STAT3 to to promote differentiation of naïve CD4 cells into Tfh cells, important mediators of humoral immunity (9). In contrast, IL6 stimulation of activated CD4 cells induces only STAT3, driving differentiation of Th17 cells that orchestrate the immune response to extra-cellular fungi (10, 11). Interestingly, STAT family members often cross-inhibit each other, and the hallmark clinical manifestation of STAT1 GOF is chronic mucocutaneous candidiasis (CMC), resulting from impaired Th17 differentiation due to partial STAT3 loss of function (12). STAT1 GOF patients frequently experience other severe invasive/opportunistic infections, and one third also develop autoimmune disease (13, 14). However, the underlying molecular and cellular mechanisms of autoimmunity in STAT1 GOF patients are unknown.

STAT1 GOF has been linked to dominant mutations in all *STAT1* domains, but most patients have mutations in the coiled-coil domain, essential for protein-protein interactions, or in the DBD, which orchestrates nuclear migration and DNA binding (13, 15, 16). Cells from STAT1 GOF patients typically show elevated pSTAT1 (Tyr701) levels after stimulation with IFNs (15), suggesting that autoimmunity could be linked to IFN hyper-responsiveness. In patients with *STAT1* DBD mutations, the most common of which is T385M (13, 17–19), STAT1 nuclear accumulation can also occur without IFN stimulation, suggesting a constitutive role for unphosphorylated STAT1 in the pathogenesis of these mutations (20). We recently showed that individuals with DBD mutations had a higher prevalence of autoimmunity compared with other STAT1 GOF patients, including endocrinopathies, autoimmune gastrointestinal disease (enteropathy or hepatitis), autoimmune cytopenia’s and interstitial lung disease (21). However, these patients also had a high rate of invasive and opportunistic infections that typically preceded autoimmunity. Thus, it is unclear whether abnormal responses to pathogenic microbes promote autoimmunity in STAT1 GOF patients with T385M and other DBD mutations. Many autoimmune disorders are more prevalent in females due to a variety of IFN-dependent and -independent mechanisms (22), so sex may also modulate autoimmunity in STAT1 GOF these patients. However, sex effects on autoimmunity have not been studied in these patients.

In addition to compromising Th17 differentiation, STAT1 GOF may also promote the differentiation of effector CD4 lineages linked to autoimmunity. T cell receptor (TCR) signals plus IFNγ-induced pSTAT1 upregulate Tbet, the regulator of Type I cytotoxic responses (23), driving differentiation of Th1 cells, implicated in inflammatory bowel disease (24) and rheumatoid arthritis (25). Th1 cells are increased in many (26, 27) yet not all (28, 29) STAT1 GOF patients. TCR signals plus IL6-induced pSTAT1 and pSTAT3 regulate early differentiation of Tfh cells, implicated in SLE and Type 1 diabetes (T1D) (30–32). Tfh cell differentiation and function is regulated by inducible T cell co-stimulator (ICOS), programmed death 1 (PD1; *Pdcd1*) and the transcriptional repressor Bcl6 (31). The transcription factors Tcf1 (*Tcf7*) and Lef1 also support Tfh differentiation (33), while restraining expression of co-inhibitory receptors such as *Ctla4* and *Lag3* (30, 33–35). Circulating Tfh-like cells (cTfh) are also increased in some STAT1 GOF patients (27, 28, 36). Foxp3^+^ T-regulatory (Treg) cells are another key subset in autoimmunity (37, 38) that range from low-normal in STAT1 GOF patients (18, 28, 29, 36, 39, 40). Thus, CD4 effector and regulatory subsets are variably affected in STAT1 GOF patients, however it remains unclear if this variability reflects specific STAT1 GOF mutation impacts and/or environmental influences such as infections or immunosuppressive treatments.

Here, we generated a mouse model to identify potential cellular, molecular and environmental mechanisms linked to autoimmunity in STAT1 GOF patients with the DBD T385M mutation. To limit the environmental impact of infection with pathogens, we studied *Stat1^T385M/+^* mice raised in a specific-pathogen-free (SPF) facility. *Stat1^T385M/+^* lymphocytes had more total Stat1 at baseline and also higher amounts of IFNγ-induced pStat1. By 15-20 weeks of age, *Stat1^T385M/+^* mice exhibited excessive splenic GC formation and multiple autoimmune manifestations compared to their co-housed *wild-type* (*WT*) littermates. High parameter immune profiling identified early and sustained expansions of splenic “memory phenotype” (MP) CD4 cells with Tfh-like features, as well as increases in activated B cells. scRNA-Seq analysis suggested that *Stat1^T385M^* activated transcription of GC-associated programs in both B and T cells, promoted aberrant activation of naïve CD4 cells and induced their differentiation into hybrid Tfh/Th1-like effector cells. Collectively, these data demonstrate that in the absence of overt infection, *Stat1^T385M^* induced aberrant B cell activation, disrupted CD4 T cell homeostasis and promoted differentiation of abnormal T helper cells, eventually leading to autoimmunity, with both processes occurring sooner and more robustly in females.

## 2 Materials and Methods

**Supplementary Tables 1–3** list key reagents and resources used in this study, sequences of the oligonucleotides, primers and repair templates used.

**Supplementary Table 2** lists vendors and catalogue numbers for key reagents and antibodies used.

**Supplementary Table 3** lists genes included in the BD Rhapsody™ Mouse Immune Response Targeted Panel.

### 2.1 Mouse husbandry

Mice were kept in a specific-pathogen free (SPF) facility (Toronto Centre for Phenogenomics; TCP) on a 12-hour light/dark cycle and provided with food and water ad-libitum. CD1 mice from Charles River (strain code: 022) and C57Bl/6J mice from JAX (strain code 000664) and TCP in-house breeding were used in this study. Mice of both sexes were used; mutants and *WT* littermates of the same sex were co-housed. Mouse procedures were carried out in compliance with the Animal for Research Act of Ontario and Guidelines of the Canadian Council on Animal Care. All procedures were reviewed and approved by the TCP Animal Care Committee, animal use protocol 25-0379H.

### 2.2 Generation of *stat1^t385m/+^* mice

A single guide RNA (sgRNA; Synthego) was used to target a protospacer adjacent motif within mouse *Stat1* exon 14. A homology-directed repair (HDR) construct (Integrated DNA Technologies) included the human *STAT1* exon 14 with containing the T385M mutation (C>T). The cassette was amplified using 5’-biotinylated primers to generate a repair template. Super-ovulated CD1 females were bred to C57Bl/6J male. Following the two-cell homologous recombination CRISPR approach (41), two-cell embryos collected at 1.5 days post-coitus were microinjected with sgRNA (50ng/μL), Cas9 conjugated with monomeric streptavidin (75ng/μL) and biotinylated repair template (20ng/μL). One day later, embryos were transferred into the oviducts of 0.5-post coitus days pseudo-pregnant CD1 females. Pups were genotyped as described below. After breeding back to C57Bl6/J, verification of congenic status was performed *via* 384-SNP background analysis (Mini Mouse Universal Genotyping Array, Transnetyx). **Supplementary Table 1** lists sequences of the oligonucleotides, primers and repair templates used.

### 2.3 Nucleic acid isolation, PCR, Sanger and whole genome sequencing

Genotyping of founder mice, as well as determination of correct splicing, were done *via* a combination of Sanger sequencing, quantitative real-time PCR (qRT-PCR) and whole genome sequencing (WSG). Founder mice underwent genomic DNA isolation followed by PCR amplification and Sanger sequencing. Samples were sequenced on Applied Biosystems SeqStudio Genetic Analyzer (Thermo Fisher). Sequencing files were analyzed using SnapGene version 5.0.8 (Insightful Science; available at snapgene.com). For splicing evaluation, RNA was extracted and reverse-transcribed, followed by qRT-PCR evaluating expression of *Stat1* transcript using QuantStudio Real-Time PCR system (Thermo Fisher). For WGS, DNA samples were extracted as described above and underwent sequencing using the Illumina NovaSeq 6000 system performed by The Centre for Applied Genomics (TCAG) at the Hospital for Sick Children. Analysis was done using Integrative Genomics Viewer (IGV) version 2.9.4 with GRCm38/mm10 as the reference genome. Routine genotyping was done *via* Sanger sequencing as described above.

### 2.4 Immunoblotting for Stat1 and pStat1

Single cell suspensions of splenocytes from 6–12 week-old mice were treated with PBS or murine recombinant IFNγ (100ng/mL) for 30 minutes at 37°CC. To assess Stat1 de-phosphorylation kinetics, splenocytes were IFNγ stimulated for 30 minutes, washed in PBS and transferred to IFNγ-free media. They were then lysed immediately (0 minutes), or cultured for an additional 60 minutes or 120 minutes in IFNγ-free media at 37°C. Cells were lysed in RIPA buffer supplemented with protease and phosphatase inhibitors. Protein lysates (10μg protein/sample) were loaded onto and separated by pre-cast polyacrylamide (4-12% Bis-Tris) gels, and then transferred to a nitrocellulose membrane using iBlot 2 Dry Blotting System (Thermo Fisher). Membranes were blocked for 1 hour at room temperature in Tris-Buffered Saline containing 5% bovine serum albumin prior to incubating overnight with anti-pSTAT1 (pY701) or anti-STAT1 plus anti-β-actin. The following day, membranes were incubated AlexaFluor-647-labelled secondary antibodies. Membranes were imaged using ChemiDoc MP imaging system (Bio-Rad) and analyzed with Image Lab software version 6.0.1 (Bio-Rad). For assessment of pStat1 phosphorylation and de-phosphorylation kinetics, pStat1 normalized to β-actin at the time of IFNγ withdrawal (0 minutes) was designated as the baseline (100%), which was compared to normalized pStat1 levels 60 and 120 minutes after withdrawal of IFNγ. **Supplementary Table 2** lists vendors and catalogue numbers for key reagents and antibodies used.

### 2.5 Measurement of serum immunoglobulin isotypes and liver panel

Sera collected from 20 week-old mice were sent to Eve Technologies (Calgary, AB) for immunoglobulin isotyping, using the Custom Mouse Immunoglobulin Isotyping 6-Plex Luminex panel (IgA, IgM, IgG1, IgG2a, IgG2b, IgG3). Liver enzymes and function, including alanine aminotransferase (ALT), aspartate aminotransferase (AST), alkaline phosphatase (ALP) and albumin were assessed in sera from 20 week-old mice by the TCP pathology core.

### 2.6 Histology and ANA detection

Pancreata collected from mice at 15-20 weeks and 49-52 weeks of age were fixed in 10% neutral-buffered formalin, sectioned, stained with hematoxylin and eosin (H&E) using standard techniques. To assess splenic architecture, spleen cryosections from 15–20 week-old mice (6 mice per genotype) were stained with H&E or with a panel consisting of GL7-FITC, CD21-TRITC, Cy5-B220 and DAPI as a nuclear counterstain. Slides were scanned using Olympus VS-120 microscope and images were analyzed using OlyVIA version 2.4 and ImageJ version 2.1.0/1.53c. These procedures were performed by the TCP pathology core. Mouse sera collected at 6-9 weeks and 15-20 weeks were screened at 1/40 for ANA using *via* indirect immunofluorescence on Hep-2 cells, according to the manufacturer’s (Bio-Rad) protocol.

### 2.7 Flow and mass cytometric immune profiling

Spleen and mLN were processed into single-cell suspensions. After washing with 1 mL staining media (SM: PBS+ 1% BSA), 1-2x10^6^ cells/sample were Fc-receptor blocked in SM at RT for 15’ prior to staining for mass or flow cytometry. For mass cytometry, cells were then stained with a 50μL cocktail of metal-tagged antibodies (**Supplementary Table 2**), washed and stained with 1 μM natural abundance Cisplatin as previously described (42). Samples were then barcoded using the Cell-ID 20-Plex PD Barcoding Kit (Fluidigm) following the manufacturer’s instructions prior to pooling up to 20 samples/tube and staining of nuclear DNA with ^191/193^Iridium. Final washes, addition 4-element EQ normalization beads and Helios acquisition were then performed as previously described (42). Helios software (v7.0.8493) was used to generate and normalize FCS 3.0 datafiles that were uploaded to CytoBank (Beckman Coulter Enterprise version) to perform sample-specific barcode stringency filtering as described (43). Additional gating removed debris, dead cells and doublets, before gating specific populations (**Figure S2**). For flow cytometry, cells were stained with 50μL of a T cell or B cell antibody cocktail (**Supplementary Table 2**) in SM (RT, 30’). After 2 PBS washes, T cell panel samples were stained with a fixable viability dye, fixed, permeabilized, and stained with anti-FoxP3 in 50μL (RT, 30’). Cells were washed, resuspended in SM and run on a 5-laser LSRFortessa analyzer running FACS-DIVA v8.0.1 (BD Biosciences). Exported FCS 3.0 files were uploaded to CytoBank, where data were compensated and pre-gated to remove debris, dead cells and doublets, prior to population gating (**Figure S3**).

### 2.8 Targeted scRNA-Seq analysis of immune gene expression

Single cell mouse splenocyte suspensions from 15 week-old female mice (3 per genotype) were stained with multiplexing antibodies, pooled, and stained with oligonucleotide-barcoded “AbSeq” antibodies. Single cells were paired with barcoded beads in a microwell cartridge using the BD Biosciences Rhapsody Express Single Cell Analysis System and Scanner, which documented retrieval of 36,059 beads with cells (8.3% multiplets). In-well single cell lysis allowed capture of RNA and AbSeq reagents bound to cells by barcoded beads prior to synthesizing cDNA and removing genomic DNA following the manufacturer’s instructions. These procedures were performed by SickKids’ Center for Advanced Single Cell Analysis (CASCA). Preparation of cDNA and AbSeq libraries followed by paired-end sequencing in an Illumina SP Nova-Seq flow cell (2x100 bp) was carried out by The Center for Genomic Analysis at the SickKids’ Research Institute.

After uploading the Fastq sequence files and Fasta mRNA+ AbSeq reference files, the SevenBridges BD Targeted Multiplex Rhapsody Analysis Pipeline (v1.10) was used to filter by read-quality prior to aligning, annotating and combining R1+R2 reads. The pipeline annotated molecules, identified putative cells and originating samples for each molecule, and output files containing expression matrices and sequencing quality metrics, yielding 6.33x10^8^ aligned mRNA reads assigned to 21,583 cells (mean=29,343 RSEC-adjusted molecule counts/cell). The RSEC_MolsperCell.csv file was uploaded to BD’s SeqGeq v1.8 where all additional analyses were performed. Briefly, data were normalized (counts/10,000 reads) and cells with low gene expression gated-out (0.4%). Of 397 mouse immune response genes analyzed (**Supplementary Table 3**), 378 were expressed at ≥100 molecules/cell in ≥ 10 cells.

Sample de-multiplixing was performed using the Lex_BDSMK Plugin (v. 0.6.0) to yield 2,810-3,734 cells/sample. Boolean “Or” gating was then used to combine samples by genotype for gating specific populations based on surface protein expression detected by AbSeq (**Figure S4**). The Seurat v.4.0.4 plugin was also used to perform unsupervised dimensionality reduction (uniform manifold approximation and projection, UMAP), multimodal (mRNA and AbSeq) clustering and identifying clusters with differentially expressed genes (mRNA) and/or proteins (AbSeq) (44). Differentially-expressed genes (DEG) between samples grouped by genotype for each population were identified using Volcano plots of fold change (FC) difference vs the negative logarithm of false-discovery rate (FDR)-adjusted q-value. The threshold for differential expression was ≥40% FC (corresponding to +/- 0.49 Log2 FC) with FDR-adjusted q-value <0.05.

The MyGeneSet tool from the Immunological Genome Project data-browser (http://rstats.immgen.org/MyGeneSet_New/index.html (45)) was used to visualize how genes over-expressed in mutant CD4 T cells were expressed in reference *WT* CD4 and CD8 T cell subsets from the microarray V1 dataset. Heatmaps were median-normalized by row (gene).

### 2.9 Statistical analysis

Statistical analyses were performed using GraphPad Prism 9 (version 9.3.1). The Shapiro-Wilk method was used to test for normal distribution of data. For normally-distributed data, unpaired two-tailed Student’s t‐tests with Welch’s correction were performed, whereas a Mann-Whitney test was performed for non-normally-distributed data, to evaluate differences between genotypes. Two-way ANOVA was used to identify the main effects of genotype versus sex as well as the interaction between these independent variables on the measured values. *Post-hoc* t-tests were performed *via* a two-stage linear step-up method of Benjamini, Krieger and Yekutieli. A false discovery rate (FDR) adjusted-p value of <0.05 represented discovery. Observed frequencies of serum ANA positivity were compared using Fisher’s exact test. Graphical data were represented as means ± standard deviation. Statistical significance was represented as: *p < 0.05, **p < 0.01, ***p < 0.001, ****p < 0.0001.

## 3 Results

### 3.1 *Stat1^T385M/+^* in mice recapitulates biochemical features of human *STAT1* GOF

**Figure 1:**
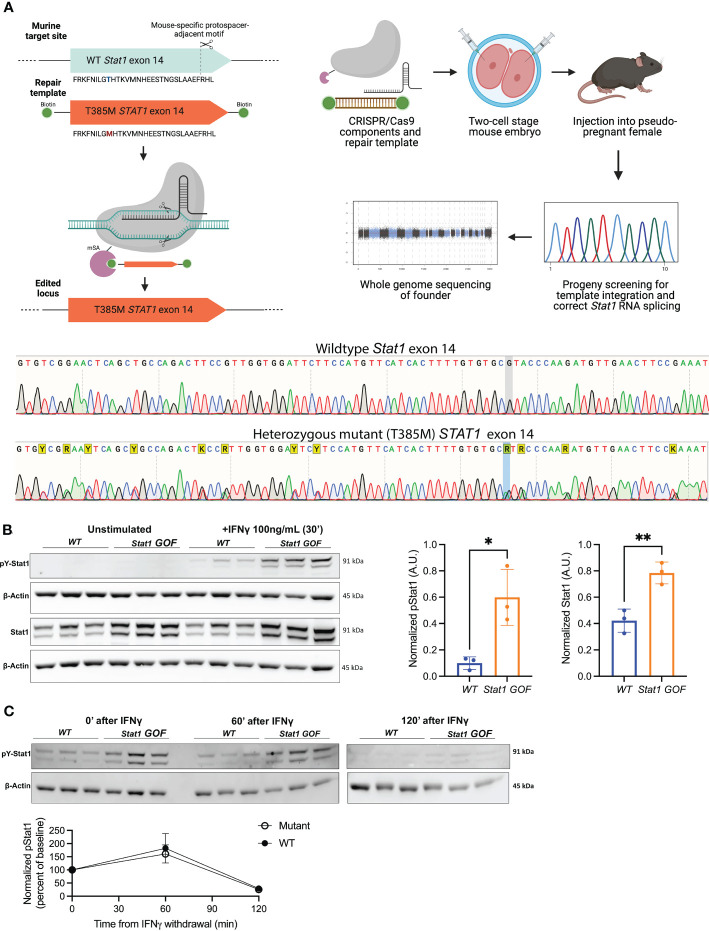
*Stat1^T385M/+^* recapitulates key biochemical hallmarks of human STAT1 gain-of-function (GOF). **(A)** *Top*: Schematic of CRISPR/Cas9 strategy used to replace *WT* murine *Stat1* exon 14 with human exon 14 harboring the T385M point mutation in the DBD. *Bottom:* Sanger sequencing confirmed the *WT* mouse exon 14 with the heterozygous mutant exon 14 in *Stat1^T385M/+^* mice. Heterozygous changes between mouse and human exon 14 are highlighted in yellow (Y: C/T; A/G; K: G/T). The T385M heterozygous point mutation on the negative strand is highlighted in blue (C>T, resulting in a G>A on the negative strand) **(B)** *Left:* Immunoblotting of splenocytes from 6–12 week-old *WT* and mutant mice (n=3/group) for total Stat1 and pStat1 (Tyr701) at baseline and after *in vitro* stimulation with 100 ng/mL IFNγ for 30 minutes. Stat1 isoforms α (91kDa) and β (84kDa) were detected.Densitometric quantification was performed on total Stat1 from unstimulated cells and on pStat1(Tyr701) from IFNγ-stimulated cells, each normalized to the β-actin loading control. Bar graphs show mean +/- SD. Unpaired 2-tailed student’s t-test with Welch’s correction: *p<0.05 (pStat1) and **p<0.01 (Stat1). **(C)** *Top:* Immunoblotting of splenocytes from *WT* and mutant mice (n=3/group) for pStat1 (Tyr701) at 0, 60 and 120 minutes following withdrawal of IFNγ stimulation (as above). *Bottom*: densitometry quantification of pStat1 (normalized to β-actin loading control) plotted as percent of starting pStat1 at time 0, immediately after IFNγ withdrawal. Unpaired 2-tailed student’s t-test with Welch’s correction: p=0.6077 (60 minutes) and p=0.6183 (120 minutes).

We used CRISPR/Cas9 technology to replace the murine *Stat1* exon 14 with the human counterpart, which introduced a C>T substitution to cause the T385M mutation in the *Stat1* DBD (**Figure 1A**) (13, 17–19). *WT* human and murine exon 14 differ by 11 base-pairs, but encode amino acid sequences which are 100% conserved between species. Exchanging the human for the mouse exon was done so that this model may be used in future studies, to test therapeutic genome-editing strategies specifically targeting the human exon 14. Splenocytes and thymocytes from *Stat1^T385M/+^* mice showed enhanced induction of pStat1(Y701) in response to acute IFNγ stimulation (**Figure 1B**). Total Stat1, both at baseline and following stimulation, was also increased as has been observed in STAT1 GOF patients (15, 46) and consistent with the known positive feedback regulation of *STAT1* (6). Importantly, de-phosphorylation in mutants was neither diminished nor delayed (**Figure 1C**), in agreement with results from STAT1 GOF patient lymphocytes (46). These data demonstrate that, similar to patients with STAT1 GOF, lymphocytes from *Stat1^T385M/+^* mice exhibited pronounced dysregulation of basal and cytokine-stimulated *Stat1* activation.

### 3.2 Spontaneous GC formation and development of age-dependent autoimmunity in *Stat1^T385M/+^* mice

**Figure 2:**
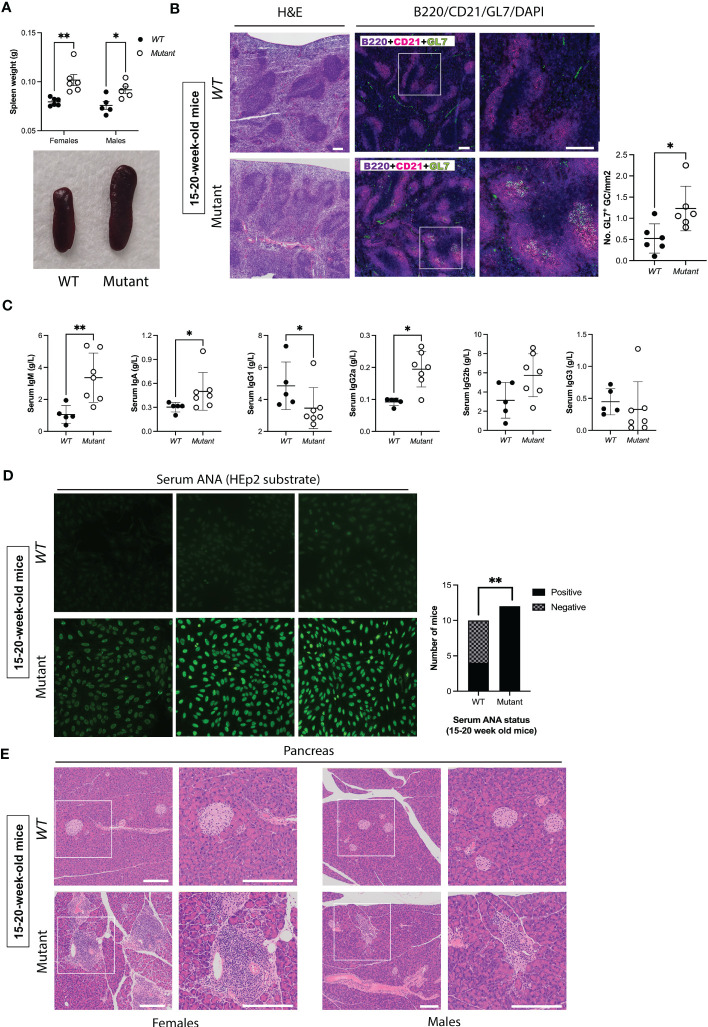
*Stat1^T385M/+^* mice develop spontaneous splenomegaly, increased germinal center formation, abnormal immunoglobulin isotype profiles and autoimmunity between 15-20 weeks of age. **(A)** Dot plots (*left*) show spleen weights in males and females, with means +/- SD indicated. Exemplar pictures are shown on the bottom. Two-way ANOVA FDR-adjusted p-values: **p<0.01 (females) vs *p<0.05 (males). **(B)** *Left*: H&E staining of spleen sections. *Middle:* Immunofluorescence staining of spleen sections with DAPI (blue), B220 (violet), CD21 (magenta) and GL7 (green). Panels on the right show area enlarged view of boxed area shown at lower magnification on the left, scale bar=200μm. *Right*: Quantification of the number of GL7^+^ germinal centers (GC)/mm^2^ per spleen (6 mice per genotype). Mann-Whitney test: *p<0.05. **(C)** Dot plots show serum levels of immunoglobulin isotypes, with means +/- SD indicated. Mann-Whitney p-values: **p<0.01 (IgM), *p<0.05 (IgA, IgG1, IgG2a), p=0.1061 (IgG2b), and p=0.2020 (IgG3). **(D)** Bar graphs (*left*) show the observed number of 20-week-old mice with negative or positive ANA detected by indirect immunofluorescence using HEp-2 cells at a screening dilution of 1:40. Fischer’s exact test **p<0.01. Exemplar images shown on the left (40X magnification) demonstrate the spectrum of fluorescence observed for each genotype, and are representative of 12/12 mutants with positive serum ANA, and 6/10 *WT* with no detectable serum ANA at this age. **(E)** Histological assessment of pancreas by hematoxylin and eosin (H&E) staining. *Left:* Exemplar images are representative of 4/4 mutant females and 0/4 *WT* females by 20 weeks of age who developed pancreatic leukocytic infiltration. *Right:* Exemplar images are representative of 3/3 mutant males and 0/4 *WT* males who developed pancreatic leukocytic infiltration by 20 weeks of age. Panels on the right show enlarged views of boxed area shown at lower magnification on the left (scale bar=200μm).

Previous mouse models of autoimmunity demonstrated splenic abnormalities including increased spontaneous GC formation (47, 48). Therefore, we evaluated spleen size and histology in roughly equal numbers of male and female mutant mice and their *WT* littermates at different ages. At 6-9 weeks of age, female but not male mutants exhibited significant splenomegaly (**Figure S1A**). However, by 15-20 weeks of age mutants of both sexes exhibited significant splenomegaly (**Figure 2A**). To evaluate architecture of B cell follicles and to assess GC formation at this age, we performed immunofluorescence using the B cell marker B220, the mature B cell/follicular dendritic cell marker CD21, and the GC B cell marker GL7. Both male and female mutants showed abnormal splenic architecture with loss of clear follicular borders. Mutant spleens also had increased density (number/mm^2^) of GL7^+^ GC, suggesting ongoing GC reactions and B cell activation (**Figure 2B**). In accordance with this notion, mutants had elevated serum levels of IgM, IgA, and IgG2a, but reduced levels of IgG1 (**Figure 2C**). These findings, and in particular the changes noted to IgG sub-classes, mirror the reported effect of enhanced IFNγ/Th1 signaling on immunoglobulin isotypes (49). Thus, by ~4 months of age, mutants displayed splenomegaly, increased GC formation and increase serum concentrations of several Ig isotypes, suggesting immune dysregulation.

To evaluate whether *Stat1^T385M/+^* mice born and raised in a SPF environment developed autoimmune manifestations, we screened sera for the presence of anti-nuclear autoantibodies (ANA) that bind HEp-2 liver cells, the gold standard method for ANA testing (50). ANA were undetectable in most mice of both genotypes between 6-9 weeks of age (**Figure S1B**). In contrast, by 15-20 weeks of age significantly more mutants were ANA^+^ compared with their *WT* littermates (**Figure 2D**). Importantly, fluorescence intensity of ANA^+^ sera was higher in mutants (**Figure 2D**), suggesting higher ANA concentrations.

We used several approaches to assess organ-specific autoimmunity. At 20 weeks of age, sera from *WT* and mutants had similar levels of liver enzymes (ALT, AST, ALP) and albumin, suggesting that mutants did not have autoimmune hepatitis (**Figure S1C**). In contrast, all mutants at this age displayed leukocytic infiltration in the pancreas manifesting as either peri-insulitis or insulitis of β-cell islets, a typical feature of T1D in mouse models (51), compared with none in *WT* (**Figure 2E**). Mutant females had higher insulitis grades at 15–20 weeks of age compared with males (**Figure 2E**), whereas mutant males developed high grade insulitis by 1 year (**Figure S1D**). Overall, these data demonstrate age-dependent and female-biased excessive spontaneous splenic GC formation and autoimmune manifestations in *Stat1^T385M/+^* mice raised in SPF conditions.

### 3.3 Expansion of PD1^+^ “memory phenotype” CD4 T cells precedes development of autoimmunity

**Figure 3:**
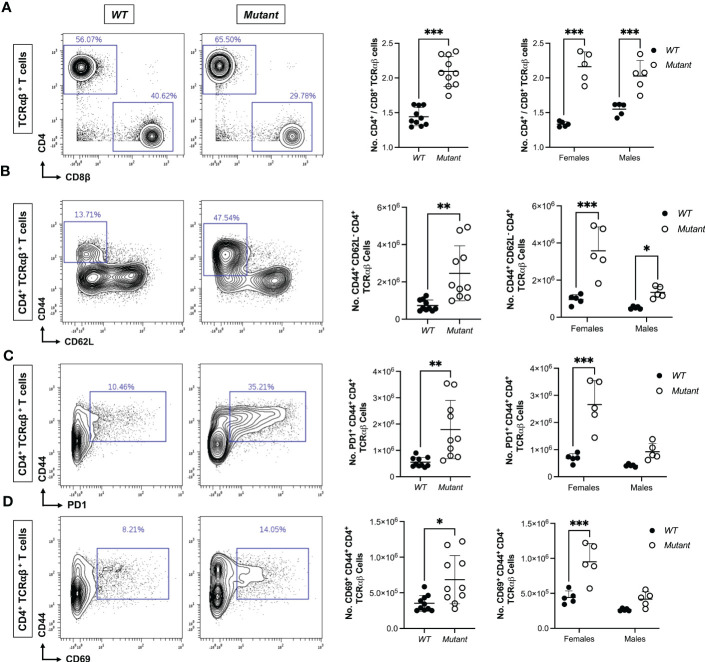
Expansion of activated splenic CD4 T cells precedes autoimmunity in *Stat1^T385M/+^* mice. Mass cytometric immune profiling of splenic T cells in 9 week-old mice. Unpaired Student’s t-test with Welch’s correction was used for all sex-aggregated genotype comparisons. Two-way ANOVA with *post-hoc* t-test was used for all genotype comparisons dis-aggregated by sex. **(A)** Left: Contour plots show CD4 vs CD8 expression gated on TCRαβ^+^ splenic T cells from exemplar mice of each strain. Right: summary dot plots show ratio of CD4^+^/CD8^+^ T cell numbers in all mice by genotype. Sex-aggregated (***p<0.001) and dis-aggregated [***p<0.001 (mutant vs. *WT* females), ***p<0.001 (mutant vs. *WT* males), interaction *p=0.03] data are shown. **(B)** Left: CD44 vs CD62L expression gated on CD4^+^ TCRαβ^+^ splenic T cells from exemplar mice of each strain. Right: summary dot plots show absolute numbers of CD44^+^ CD62L^-^ (MP) CD4^+^ T cells in all mice by genotype. Sex-aggregated (**p<0.01) and dis-aggregated [***p<0.001 (mutant vs. *WT* females), *p<0.05 (mutant vs. *WT* males), interaction **p=0.009] data are shown. **(C)** Left: Contour plots show CD44 vs PD1 expression gated on CD4^+^ TCRαβ^+^ splenic T cells from exemplar mice of each strain. Right: summary dot plots show absolute numbers of PD1^+^ CD44^+^ CD4^+^ T cells in all mice by genotype. Sex-aggregated (**p<0.01) and dis-aggregated [***p<0.001 (mutant vs. *WT* females), P=0.11 (mutant vs. *WT* males), interaction **p=0.003] data are shown. **(D)** Left: Contour plots show CD44 vs CD69 expression gated on CD4^+^ TCRαβ^+^ splenic T cells from exemplar mice of each strain. Right: summary dot plots show absolute numbers of CD69^+^ CD44^+^ CD4^+^ T cells in all mice by genotype. Sex-aggregated (*p<0.05) and dis-aggregated [***p<0.001 (mutant vs. *WT* females), P=0.07 (mutant vs. *WT* males), interaction *p=0.02] data are shown. For all dot plots, mean +/- SD are presented.

To identify immune abnormalities that precede development of frank autoimmunity at 15-20 weeks of age, we used mass cytometry to profile immune cell lineages and activation states in spleen and mesenteric lymph nodes (LN) from a 9-week-old cohort of *Stat1^T385M/+^* and *WT* littermates. *WT* and mutant spleens had similar numbers of B, NK, and most myeloid cell populations, except for a small increase in macrophages in mutants (**Figures S2A–D**). Although splenic T cell numbers were similar in mutant versus *WT* mice (**Figure S2A**), the ratios of CD4/CD8 T cell number were significantly higher in mutant females and males (**Figure 3A**), reflecting significantly more CD44^+^ CD62L^-^ CD4 cells in mutants (**Figure 3B**). These non-naïve “memory phenotype” (MP) cells, also known as “natural memory” T cells, develop in unimmunized mice under SPF conditions in response to low levels of tonic TCR and cytokine signaling (52). Interestingly, more mutant MP CD4 cells expressed PD1, a co-inhibitory receptor that marks the Tfh sub-lineage of CD4 T cells (**Figure 3C**), and the activation marker CD69 (**Figure 3D**), suggesting that mutant MP CD4 T cells were more activated. Importantly, numbers of PD1^+^ CD44^+^ and CD69^+^ CD44^+^ CD4 T cells were significantly increased only in female mutants (**Figures 3C, D**). Mutants also had more PD1^+^ MP CD4 T cells in mesenteric LN, but the increase was less pronounced than in spleen (data not shown). In contrast to these significant impacts of *Stat1^T385M^* on CD4 T cells, PD1 was not elevated in mutant CD8 T cells and they had similar numbers of MP CD8 T cells (defined as CD44^+^ CD122^+^) as *WT* (**Figure S2E** and data not shown). In addition, 2-way ANOVA showed that sex significantly modified the impact of genotype on the abundance of MP CD44^+^ CD62L^-^ CD4 cells (interaction P=0.009). These data demonstrate that an early, selective expansion of PD1^+^ MP CD4 T cells precedes the development of frank autoimmunity in both sexes, but in a more variable and robust fashion in females.

### 3.4 Expansion of ICOS^+^ Tfh-like cells and activated B cells in 15-week-old mutant mice

**Figure 4:**
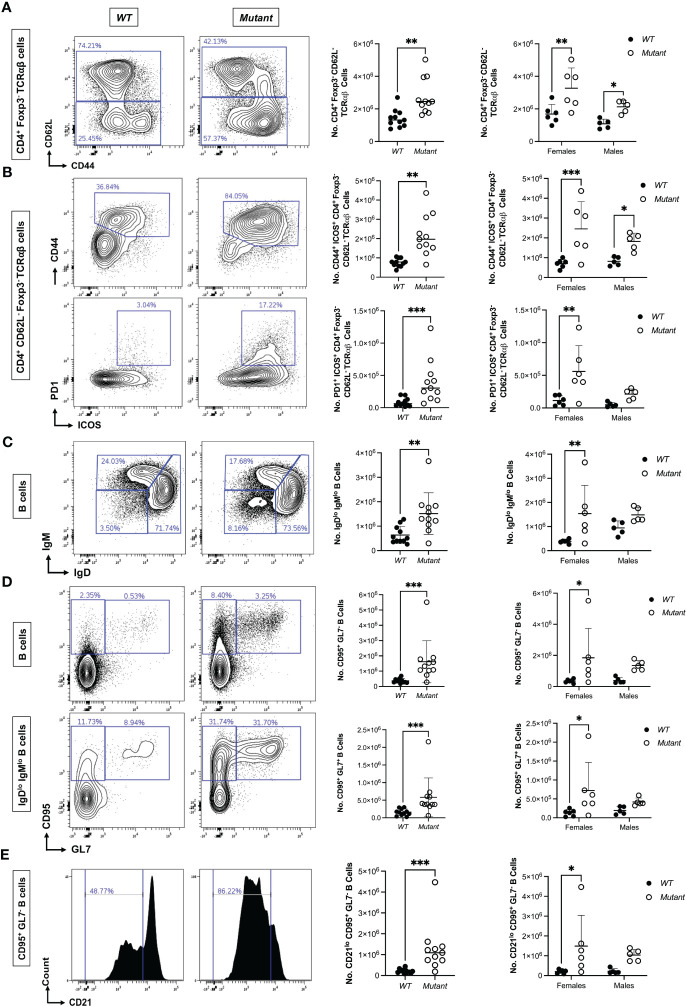
Expansion of ICOS^+^ PD1^+^ Tfh-like MP CD4 cells and activated B cells in 15 week-old *Stat1^T385M/+^* mice. Flow cytometric immune profiling of splenic T cells in 15 week-old mice. Unpaired student’s t-test with Welch’s correction was used for all sex-aggregated genotype comparisons except where Mann-Whitney tests are indicated below. Two-way ANOVA with *post-hoc* t-test was used for all genotype comparisons dis-aggregated by sex. **(A)** *Left:* Exemplar contour plots of CD44 vs CD62L expression gated on CD4^+^ Foxp3^-^ TCRαβ^+^ splenic T cells from each strain. *Right*: Summary dot plots of CD62L^-^ CD4^+^ Foxp3^-^ T cell numbers per spleen by genotype. Sex-aggregated (Mann-Whitney **p<0.01) and dis-aggregated [**p<0.01 (females) vs *p<0.05 (males), interaction p=0.42] data are shown. **(B)** *Top left*: Exemplar contour plots of CD44 vs ICOS expression gated on CD4^+^ Foxp3^-^ CD62L^-^ TCRαβ^+^ splenic T cells from each strain. *Bottom left:* Exemplar contour plots of PD1 vs ICOS expression gated on CD4^+^ Foxp3^-^ CD62L^-^ TCRαβ^+^ splenic T cells from each strain. *Top right*: Summary dot plots of numbers of ICOS^+^ CD44^+^ CD62L^-^ CD4 cells per spleen by genotype. Sex-aggregated (**p<0.01) and dis-aggregated [***p<0.001 (females) vs *p<0.05 (males), interaction p=0.27] data are shown. *Bottom right*: Summary dot plots of numbers of ICOS^+^ PD1^+^ CD62L^-^ CD4 cells per spleen by genotype. Sex-aggregated (Mann-Whitey ***p<0.001) and dis-aggregated [**p<0.01 (females) vs p=0.13 (males), interaction p=0.14] data are shown. **(C)** *Left*: Exemplar contour plots of IgM vs IgD gated on CD19^+^ splenic B cells from each strain. *Right*: Summary dot plots of IgM^lo^ IgD^lo^ B cell numbers per spleen by genotype. Sex-aggregated (**p<0.01) and dis-aggregated [**p<0.01 (females) vs p=0.11 (males), interaction p=0.29] data are shown. **(D)** *Left*: Exemplar contour plots of CD95 vs GL7 expression gated on either total (*top*) or IgM^lo^ IgD^lo^ (*bottom*) splenic B cells. *Right*: Summary dot plots of absolute number of activated CD95^+^ GL7^-^ (top) or CD95^+^ GL7^+^ (bottom) B cells per spleen. For CD95^+^ GL7^-^ B cells, sex-aggregated (Mann-Whitney ***p<0.001) and dis-aggregated [*p<0.05 (females) vs p=0.08 (males), interaction p=0.55] data are shown. For CD95^+^ GL7^+^ B cells, sex-aggregated (Mann-Whitney ***p<0.001) and dis-aggregated [*p<0.05 (females) vs p=0.2 (males), interaction p=0.3] data are shown. **(E)** *Left*: Exemplar histogram of CD21 gated on CD95^+^ GL7^-^ splenic B cells. *Right*: Summary dot plots of CD21^lo^ CD95^+^ GL7^-^ B cell numbers in all mice by genotype. Sex-aggregated (Mann-Whitney ***p<0.001) and dis-aggregated [*p<0.05 (females) vs p=0.07 (males), interaction p=0.52] data are shown. For all dot plots, mean +/- SD are presented.

Since *Stat1^T385M/+^* mice exhibited autoimmune manifestations by 15-20 weeks of age, we performed immune profiling on another cohort of *WT* and mutant mice aged to 15 weeks, adding additional markers for Tfh (ICOS) and T regulatory (FoxP3) CD4 cells. The ratio of CD4/CD8 T cell number was again increased, but the number of Foxp3^+^ CD4 T cells was similar to *WT* (**Figures S3A, B**), revealing no impact of *Stat1^T385M^* on abundance of this important immunosuppressive subset. In contrast, mutants had more non-naïve CD62L^-^ Foxp3^-^ CD4 T cells (**Figure 4A**). Within this population, mutants had significantly more CD44^+^ ICOS^+^ cells (**Figure 4B**) and more PD1^+^ ICOS^+^ cells (**Figure 4B**), suggesting expansion of Tfh-like cells. Similar to our findings in 9 week-old mice, T cell differences in 15 week-old mice were generally more significant but also more variable in females (**Figures 4A, B**). T cell changes in mLN were similar to those identified in spleen, though slightly less robust (**Figure S3C**).

Tfh cells also promote differentiation and isotype class-switching in GC (31), and our histological analysis revealed increased GC B cells in 15-20 week-old mice (**Figure 2B**). Therefore, we profiled B cell differentiation states using CD21, IgM, IgD, CD95 and GL7. *WT* and mutant mice had similar numbers of total B cells, as well as classically defined follicular, marginal zone and transitional B cells (**Figure S3D**). However, mutants had more IgM^lo^ IgD^lo^ class-switched and more activated CD95^+^ and CD95^+^ GL7^+^ GC B cells in the spleen (**Figures 4C, D**) and mLN (**Figure S3E**). Mutants also had more splenic CD21^lo^ CD95^+^ GL7^-^ B cells (**Figure 4E**), a phenotype associated with Tbet^+^ B cells found in certain autoimmune conditions in humans (53). Both CD95^+^ subsets were greatly enriched among IgM^lo^ IgD^lo^ B cells, suggesting that in mutants, there were more activated B cells engaged in class-switching. Interestingly, in contrast to the T cell data, when the B cell data were dis-aggregated by sex the *Stat1^T385M^* impacts were only significant in females (**Figures 4C–E**). However, similar to the T cell data female mutants again showed greater variability than male mutants. Thus at 15 weeks of age, while both sexes exhibited significant expansion of Tfh-like MP CD4 cells, only female *Stat1^T385M/+^* mice exhibited a significant increase in activated and class-switched B cells.

Collectively, these immune profiling experiments revealed that *Stat1^T385M/+^* mice raised in SPF conditions exhibit early and prolonged expansion of Tfh-like MP CD4 in both sexes. These effects were more robust on average in females but also more variable and were seen in spleen and mLN. The *Stat1^T385M^* impacts on B cells were not evident until 15 weeks of age and were restricted to females at this timepoint.

### 3.5 *Stat1^T385M^* enhances CD4 T cell activation and imparts Tfh-like and Th1-like effector programs

**Figure 5:**
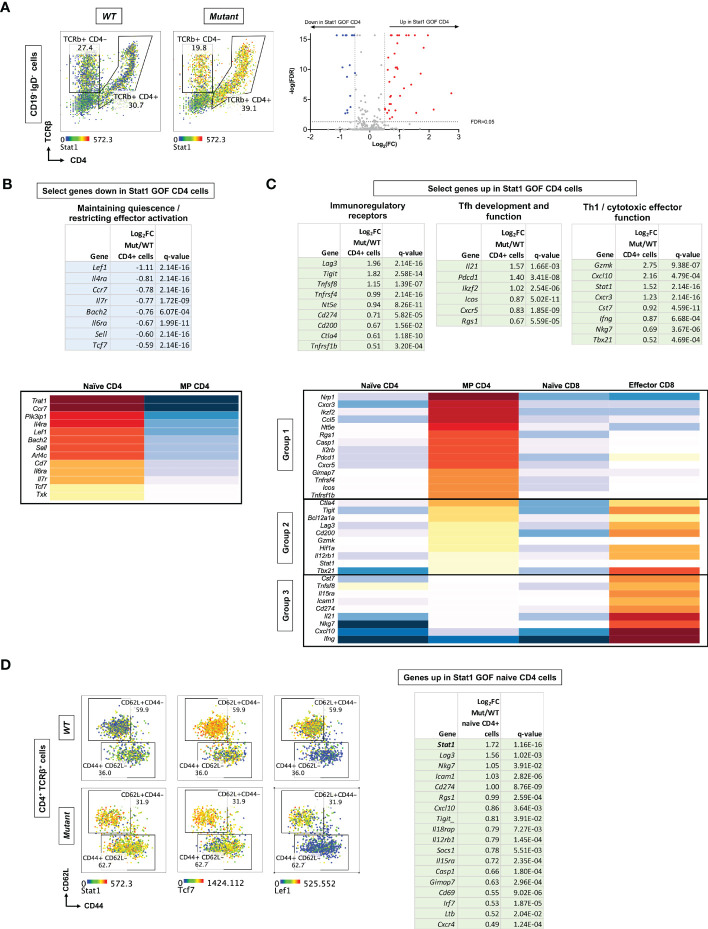
scRNA-Seq profiling of immune gene expression in *WT* versus *Stat1^T385M/+^* spleen cells from 15 week-old mice. **(A)** (*Left*): Dot plots show CD4 and TCRβ expression on CD19^-^ IgD^-^ cells, defining CD4 T cells (CD4^+^ TCRβ^+^) and CD8 T cells (CD4^-^ TCRβ^+^ expressing *Cd8b1-* see **Figure S4D**). Overlaid heatmap shows *Stat1* expression in CD4 and CD8 T cells. (*Right*): Volcano plots show DEG between mutant and *WT* CD4 T cells, plotted according to Log_2_fold change [Log_2_FC (mutant/*WT*)], against the negative logarithmic value of the false discovery rate (-logFDR). Genes up-regulated or down-regulated in mutant are denoted in red and blue, respectively. (B, C) *Top:* Tables show select genes down-regulated **(B)** or up-regulated **(C)** in mutant CD4 T cells by 40% or more. Gene expression tables show Log_2_FC values and FDR-adjusted q-values <0.05. *Bottom:* Heatmap visualization of genes down-regulated **(B)** or up-regulated **(C)** in mutant CD4 cells in selected splenic T cell reference populations from the Immunological Genome Project (45): naïve CD4 or CD8 cells, MP CD4 cells and effector CD8 cells (isolated 12h following infection with *Listeria*). Upregulated genes were grouped (1–3) according to their pattern of expression in the reference populations. **(D)** *Left:* Dot plots show CD44 vs CD62L expression on CD4^+^ TCRβ^+^ cells in mutants and *WT*, distinguishing naïve CD62L^+^CD44^-^ from MP CD44^+^ CD62L^-^ CD4 cells. Expression levels of genes of interest are overlaid as heatmaps. *Right:* Table shows genes upregulated in mutant naive CD4 T cells by 40% or more. FC values are presented along with FDR-adjusted q-values <0.05.

To elucidate the transcriptional impact of *Stat1^T385M^* in T and B cells, we performed targeted scRNA-seq analysis of splenocytes from 15 week-old *WT* and mutant (n=3/group), quantifying transcripts encoding 397 immune genes as well as surface expression of 10 proteins detected with oligo-nucleotide tagged “AbSeq” antibodies. After de-multiplexing, we gated on CD19^+^ IgD^+^ B cells, TCRβ^+^ CD4^+^ and TCRβ^+^ CD4^-^ T cell subsets (**Figure S4A**). Importantly, mutants in this small cohort had higher ratios of CD4/CD8 T cells and of CD62L^-^/CD62L^+^ cells within the CD4 T cell compartment (**Figure S4B**), similar to flow cytometric profiling of the larger cohort reported above. We then merged the individual samples from each group to identify genes whose expression differed significantly (≥40% with FDR-adjusted q-value of <0.05) between *WT* vs mutant in each of the 3 subsets. *Stat1* itself was among the top DEG and was over-expressed in all mutant lymphocyte populations (**Figure 5A**; **Figures S4C, D**, **Tables S1–S3**), in keeping with its known capacity to positively regulate its own expression (6). There were more over-expressed than under-expressed genes in mutants relative to *WT*, suggesting that *Stat1^T385M^* acted primarily as a transcriptional activator in all lineages. Interestingly, *Stat1* was more highly upregulated in mutant T cells relative to mutant B cells. CD4 T cells had ~3-4 times as many DEG as CD8 T cells or B cells and most were over-expressed in mutants (n=33; **Figure 5A**), suggesting that *Stat1^T385M^* had the greatest transcriptional impact on CD4 T cells.

Among the genes significantly down-regulated in mutant CD4 T cells were several associated with a naïve quiescent state (54, 55), such as *Sell* (encoding CD62L), *Ccr7, Il6ra, Il7ra*, and *Bach2* (**Figure 5B** top), suggesting that they were more activated. This group also included *Lef1* and *Tcf7*, which restrain induction of co-inhibitory receptors and promote expression of Tfh genes during anti-virus responses (33–35); *Tcf7* also restrains induction of cytotoxicity genes in CD8 T cells (55). Conversely, mutant CD4 T cells significantly over-expressed several genes encoding immunoregulatory receptors known to be induced during T cell activation (56) (**Figure 5C** top), including *Tnfrsf4* (encoding Ox40), *Tnfsfr1b* (encoding Tnfr2), *Pdcd1*, *Lag3, Cd200, Tigit, Ctla4* and *Nt5e* (encoding CD73). Mutant CD4 T cells also over-expressed the Tfh-associated genes *Icos, Cxcr5, Il21 and Ikzf2* (31, 57, 58); however, *Bcl6*, the master Tfh regulator (31), was not differentially expressed. Several cytotoxicity/Th1-associated molecules were also overexpressed: *Tbx21* encoding Tbet*, Ifng, Cst7, Nkg7* and *Gzmk* (**Figure 5C** top). This signature suggested that *Stat1^T385M^* may enhance CD4 T cell activation while also imparting both Tfh-like and Th1-like effector programs.

To better understand the unusual differentiation state of CD4 T cells from *Stat1^T385M/+^* mice, we visualized how the DEG were expressed by reference *WT* CD4 and CD8 T cell populations profiled by the Immunological Genome Project (45). Notably, all genes expressed at lower levels in mutant CD4 cells were more highly expressed by reference naïve compared to reference MP CD4 cells (**Figure 5B**, bottom). This finding confirms that *Stat1^T385M^* downregulates genes associated with the naïve state of normal CD4 T cells. We also visualized how genes up-regulated in mutant CD4 T cells were expressed in reference naïve and MP CD4 subsets. We added a reference effector CD8 subset, since we had observed several cytotoxicity-associated genes among those upregulated by *Stat1^T385M^*. Interestingly, the *Stat1^T385M^*-upregulated genes fell into 3 groups (**Figure 5C**, bottom). Group 1 genes were most highly expressed by reference MP CD4 cells, and included *Nrp1, Icos, Pdcd1 and Cxcr5*, suggesting that these Tfh-associated genes are also up-regulated as part of the natural CD4 memory program. Group 2 genes were highly expressed by reference MP CD4 and effector CD8 cells, and included *Ctla4, Lag3, Tigit, Stat1, Tbx21* and *Gzmk.* Finally, Group 3 genes were most highly expressed by reference effector CD8 cells, and included *Cst7*, *Nkg7*, *Ifng, Cxcl10, Il21, Il15ra* and *Cd274* (encoding PD-L1). This analysis confirmed that mutant CD4 T cells over-express Tfh-associated and other genes that distinguish *WT* naïve from MP CD4 cells, but they also expressed genes associated with cytotoxic differentiation.

To determine which aspects of the *Stat1^T385M^*-associated gene expression program identified above were already present in the naïve state, we performed DEG analysis comparing WT vs mutant naïve CD62L^+^ CD44^-^ as well as MP CD44^+^ CD62L^-^ CD4 cells (**Figure 5D**; **Tables S4, S5**). Interestingly, *Stat1* was the most highly over-expressed gene in mutant naive CD4 T cells, which also over-expressed certain activation (*Lag3, Tigit, Cd69, Icam1*), IFN-regulated (*Cd274, Socs1, Cxcl10*), cytokine receptor (*Il12rb1* and *Il15ra*) and cytotoxicity (*Nkg7*) genes (**Figure 5D**; **Table S2**). Indeed, several genes belonging to each of the 3 Groups of up-regulated genes identified in total CD4 cells (**Figure 5C**) were overexpressed specifically in mutant naive CD4 cells. As expected, many of the up-regulated genes in total mutant CD4 cells were also up-regulated specifically in mutant MP CD4 cells (**Table S4**). Additionally, mutant MP CD4 cells had decreased expression of *Ccr6* and *Rorc* (encoding RORγt), suggesting that *Stat1^T385M^* inhibited Stat3 activation and impaired Th17 effector differentiation, as previously observed in STAT1 GOF patients (15). Other lineage-defining transcription factors (*Gata3, Foxp3*) were not differentially expressed between *WT* and mutants.

**Figure 6:**
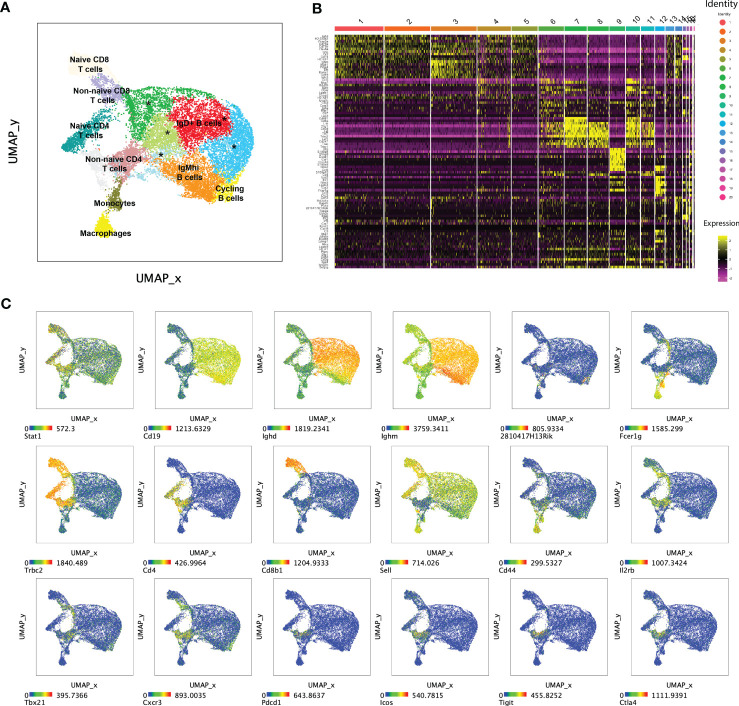
Seurat multi-modal clustering of 15 week-old mouse splenocytes. **(A)** Plot shows uniform manifold approximation and projection (UMAP) dimensionality reduction overlayed with Seurat-generated clusters on aggregated WT and mutant cells. Clusters marked with an asterisk (*) represent IgD+ B cells. **(B)** Heatmap shows relative expression of Seurat-defined “marker” genes across 20 Seurat-generated clusters. **(C)** Dot plots show UMAP of aggregated WT and mutant cells colored by expression levels of informative transcripts. Note that 2810417H13Rik encodes Pclaf (PCNA clamp associated factor), a cell cycle regulatory gene.

We also used Seurat to perform unsupervised multimodal (mRNA and AbSeq) dimensionality reduction and clustering to ask whether mutants contained T or B cell subsets with unique immune gene profiles. Among the 14 clusters with >100 cells in merged *WT* or mutant samples, there were two myeloid (macrophages and monocytes), seven B cell and five T cell clusters that were all present in both *WT* and mutant samples (**Figures 6A, B** and **Table S6A**). Thus, targeted single cell profiling of immune gene and protein expression by total splenocytes identified multiple clusters of naïve and activated/effector of T and B cells. However, mutant-specific T or B cell clusters were not detected, suggesting that *Stat1^T385M^* did not promote differentiation of a trancriptionally unique T or B cell effector subset.

All mutant B cell clusters expressed more *Stat1* than their *WT* counterparts (**Figure 6C**; **Table S6C**). Five of seven also over-expressed *Tbx21* and/or its target *Cxcr3*; both are known to regulate B cell proliferation and migration to the GC dark zone during malaria infection (59). Indeed, several mutant B cell clusters over-expressed one or more GC-associated genes such as *Fas/CD95* (60)*, Irf8* (61) and *Icam1* (62), as well as *Igh* isotypes that result from class-switching. A similar set of GC-associated genes were also up-regulated in our comparison of total mutant versus *WT* B cells (**Table S3**). GC B cells are highly proliferative, but high levels of cell cycle-associated genes such as *Pclaf, Mki67* and *Pcna*, were restricted to a single small B cell cluster (#14), which was more abundant in mutants (**Figure 6C**; **Table S6B**). This unsupervised analysis showed that *Stat1^T385M^* activated aspects of the GC B cell program, though not *Bcl6*, across multiple B cell subsets.

Among the five T cell clusters, Seurat identified *Sell^+^* (encoding CD62L*)* naïve and *Sell^-^* non-naïve CD4 and CD8 T cell subsets. A comparison of mutant/*WT* CD8 clusters yielded a small number of DEG highly similar to those identified in our analysis of total CD8 T cells, with the addition of *Gzmk* in naïve CD8 cells (**Figure S4**). The two non-naïve CD4 T cell clusters (6 and 11) expressed high levels of *Il2rb*, *Tbx21, Cxcr3*, together with the Tfh-associated genes *Pdcd1, Icos, Ctal4* and *Tigit*, suggesting a hybrid Tfh/Th1 program (**Figure 6C**). Mutant clusters 6 and 11 expressed higher levels of these genes and/or proteins together with more *Stat1*, *Gzmk, Cxcl10, Il21, Lag3, Nt5e* and *Tnfrsf4* to name a few (**Table S6C**). Overall, unsupervised and immunophenotype-guided analysis of single cell immune gene expression identified highly overlapping sets of DEG which collectively suggested that *Stat1^T385M^* activated transcription of GC-associated programs in both B and T cells. However, *Stat1^T385M^* had the strongest transcriptional impact on T cells, promoting aberrant activation of naïve CD4 cells and inducing their differentiation into hybrid Tfh/Th1-like effector cells.

## 4 Discussion

We generated and characterized *Stat1^T385M/+^* mice to model the impact of the DBD class of STAT1 GOF mutations on immune homeostasis under SPF conditions in both males and females. Similar to patients with STAT1 GOF (46), *Stat1^T385M/+^* lymphocytes had more total Stat1 at baseline and also higher amounts of IFNγ-induced pStat1. By 9 weeks of age, *Stat1^T385M/+^* mice showed expansion of MP CD4 cells expressing Tfh-like markers, in spleen and in gut-draining mLN, with no observed autoimmunity. By 15-20 weeks of age mutants also displayed B cell activation, increased GC formation, abnormal immunoglobulin isotype profiles and development of autoimmunity. Notably, females developed these immune abnormalities sooner and more robustly than males, identifying significant sex effects for this mouse model of STAT1 DBD GOF. Single cell analysis showed that although *Stat1* was significantly up-regulated in both T and B cells from *Stat1^T385M^* mutants, its most predominant impact was to promote aberrant activation of naïve CD4 cells and induce their differentiation into hybrid Tfh/Th1-like effector cells. Collectively, these data demonstrate that *Stat1^T385M^* increased basal and cytokine-stimulated Stat1 activation in lymphocytes, disrupting naïve CD4 T cell homeostasis and promoting differentiation of abnormal T helper cells with cytotoxic features (63) as well as GC-like B cells, eventually resulting in autoimmunity in the absence of overt infection. These findings potentially explain how human STAT1 GOF causes immune dysregulation and autoimmunity.

The biochemical, immunophenotypic and overt autoimmune abnormalities we observed in *Stat1^T385M/+^* mice differ from previously published studies modeling the immune impacts of STAT1 GOF mutations in the coiled-coil domain. In *Stat1^R274W/+^* (64) mice, total Stat1 was not increased at baseline, and IFN-induced Stat1 hyper-phosphorylation was noted in T cells; however T cell numbers were normal (64). *Stat1^R274Q/+^* mice also showed increased IFN-stimulated pStat1 in gut CD4^+^ T-cells (65). However, autoimmunity was not reported in either model, and viral or fungal infection was required to induce overt immunophenotypic or transcriptional changes in both. In contrast, splenocytes from our *Stat1^T385M/+^* mice had more total Stat1 at baseline and in IFNγ-induced pStat1 but normal Stat1 dephosphorylation kinetics, all in keeping with observations made in patient cells (46). The immunophenotypic and transcriptional profiles of *Stat1^T385M/+^* mice were also significantly different from *WT* in our SPF colony. These findings suggest that there is differential pathogenicity of STAT1 GOF mutations; the T385M DBD mutation selectively disrupted immune homeostasis and promoted autoimmunity in the absence of deliberate stimulation or infection.

Here we report significant sex effects for both the kinetics and magnitude of immune abnormalities in our *Stat1^T385M/+^* GOF mouse model. Female mutants developed splenomegaly earlier, and also displayed more robust increases in MP CD4 cells between 9-15 weeks. These cells also expressed significantly higher amounts of PD1 and ICOS, suggesting that MP CD4 cells were more activated than in males. By 15 weeks, activated IgD^lo^ IgM^lo^ GC B cells were significantly elevated only in females. In contrast, studies of humans or other mouse models with STAT1 GOF did not report sex effects on development of immune abnormalities and autoimmunity. Most human autoimmune diseases preferentially affect females due to a combination of factors, including increased expression of X-linked innate and adaptive immune genes, heightened type I IFN secretion mediated by estrogens, and microbiome differences (22, 66). Additionally, *STAT1* contains an estrogen-response element, suggesting an IFN-independent role for estrogens in STAT1 activation (67). However, sex effects identified in mice do not always apply to humans. For instance, while female mice show a higher predisposition than males to developing T1D, whereas humans of both sexes develop T1D in nearly equal ratios (22). Investigation of the possible sex effect of autoimmunity in human STAT1 DBD GOF is therefore warranted, including assessment of the types of autoimmune manifestation, age of onset and severity.

*Stat1^T385M^* had the greatest immunophenotypic impact on MP CD4 T cells, which in *WT* mice, differentiate in response to homeostatic TCR signaling and cytokine stimulation. MP CD4 T cells are generated in mice free of all microbes and exogenous food antigens (52), suggesting that they differentiate cell autonomously and/or in response to self-antigens. While we cannot rule out the contribution of commensal microbes to the immune changes observed in our SPF *Stat1^T385M/+^* mice, these changes were not more prominent in gut-draining mLN, as would be expected if commensal gut microbiota drove CD4 T cell activation. Further studies will be required to determine the role of self- versus foreign- antigens as well TCR and cytokine signaling in driving aberrant MP CD4 T cell differentiation in this mouse model of STAT1 DBD GOF.

*Stat1^T385M^* had also the greatest transcriptional impact on CD4 T cells, which over-expressed several Tfh-associated genes a well as immunoregulatory receptors associated with Tfh and also with chronic T cell activation and exhaustion. Elevated expression of co-inhibitory activation markers, such as PD-L1 and Fas was previously reported in STAT1 GOF patient CD4 T cells, and was suggested as a mechanism potentially leading to T cell exhaustion over time (39, 68, 69). Although *Stat1^T385M^* MP CD4 T cells did not over-express *Bcl6*, which encodes the master regulator of Tfh differentiation (70), they over-expressed several other Tfh-associated genes such as *Icos, Pdcd1, Icam1, Ikzf2, Il21*and *Cxcr5*, suggesting that *Bcl6* up-regulation is not required to induce these genes when Stat1 is activated. Similar to early Tfh cells, mutant CD4 T cells also expressed *Il21* but not *Il4*; the latter cytokine is made only by fully differentiated Tfh cells (31). IL21 together with other signals can drive B cell proliferation and differentiation both within and outside of the GC. Therefore, it seems likely that, in addition to a primary impact of *Stat1^T385M^* on B cells, the expanded Tfh-like cells may also have contributed to the increase in activated B cells and excessive splenic GC formation. Interestingly, T cell-intrinsic overproduction of IFNγ also causes accumulation of Tfh cells, GC B cell activation and SLE (71), phenomena that are likely linked to Stat1 activation. Our scRNA-Seq based immune gene profiling showed that in addition to early Tfh-like features, *Stat1^T385M^* CD4 T cells also expressed a cytotoxic CD8/Th1-like effector program. Although multiple lines of evidence show that Bcl6 and Tbet cross inhibit each other, infection-induced Tfh cells can transiently express Tbet and Th1 features (70, 72–75). Thus, STAT1 GOF mimics some aspects of infection-induced Tfh differentiation. During acute viral infection, IL6 activates both Stat1 and Stat3, with the latter being required to limit IL2/Stat5-induced Th1 differentiation (9, 76, 77). However, this break on Th1 differentiation may be impaired in *Stat1^T385M^* CD4 T cells since activated Stat1 inhibits Stat3. Peripheral blood from STAT1 GOF patients shows variable in Th1 cells, circulating Tfh (cTfh) cells as well as cTfh with “Th1-like” features (such as increased Tbet and CXCR3 expression) (26–28, 36, 78). Thus, our *Stat1^T385M/+^* mouse model recapitulates several features of abnormal CD4 T cell differentiation seen in STAT1 GOF patients, but in the absence of infection.

Interestingly, naïve CD4 T cells highly over-expressed *Stat1* and also displayed some aspects of the *Stat1^T385M^*-driven abnormal program, which became more prominent in MP CD4 T cells. However, these phenotypically naïve cells also exhibited unique transcriptional changes, likely setting the stage for abnormal differentiation. They overexpressed *Il2rb* and *Il15ra*, cytokine receptors that drive differentiation of MP CD4 cells (52). Notably, mutant CD4 T cells expressed significantly lower amounts of *Tcf7* and *Lef1*, transcription factors which restrain expression of the *Ctla4* and *Lag3* co-inhibitory receptors in Tfh cells (35), providing a potential mechanism for over-expression of these genes in mutant CD4 T cells. *Tcf7* and *Lef1* also restrain expression of cytotoxicity genes in naïve CD8 cells (55), so their downregulation, together with Stat1 GOF, could contribute to the up-regulation of a Type I/cytotoxicity program marked by expression of *Tbx21, Gzmk, Nkg7, Cst7*, *Cxcr3* and *Cxcl10. Tcf7* and *Lef1* also critically regulate Tfh differentiation and *Bcl6* induction during infection-induced Tfh differentiation (33, 35), so their *Stat1^T385M^*-induced down-regulation could contribute to the lack of *Bcl6* expression by mutant Tfh-like CD4 cells. *Stat1^T385M^*-induced *Tbx21* could also contribute to the lack of *Bcl6* expression. While the precise molecular mechanisms remain to be elucidated, our findings suggest that *Stat1^T338M^* alters expression of key transcription factors to aberrantly activate naïve CD4 T and promote their differentiation into hybrid Tfh/Th1-like CD4 cells under homeostatic conditions, ultimately promoting autoimmunity in the absence of infection.

## Data availability statement

The datasets presented in this study can be found in online repositories. The names of the repository/repositories and accession number(s) can be found below: PRJNA949654 (SRA).

## Ethics statement

The animal study was reviewed and approved by The Centre for Phenogemonics Animal Care Committee.

## Author contributions

Study conceptualization by OS, CG, CR, EA, RC. Methodology by OS, SV, BG, CG. Investigation by OS, SV, ER, DM, CG. Data analysis by OS, CG. Visualization by OS, CG. Supervision by RC, CR, CG and EI. Funding acquisition by OS, CR, RC. Writing - original draft by OS. Writing – review and editing by OS, SV, ER, DM, BG, CR, DC, CG, EI. All authors contributed to the article and approved the submitted version.

## Supplementary material

Supplementary Figure 1 Autoimmune manifestations in Stat1T385M/+ mice from 6 weeks to 1 year of age. (A) Dot plots show spleen weights in 9-week-old males and females, with means +/- SD indicated. Two-way ANOVA: FDR-adjusted p-values: *p<0.05 (females) vs p=0.42 (males). (B) Bar graphs show the observed frequency of serum positivity for anti-nuclear antibodies (ANA) as detected in 6-9-week-old mice via indirect immunofluorescence using HEp-2 substrate, at a screening dilution of 1:40. Fischer’s exact test **p>0.999. (C) Dot plots show serum values of liver parameters including alanine amino transferase (ALT), aspartate aminotransferase (AST), alkaline phosphatase (ALP) and albumin in 20 week-old mutants and *WT* littermates, with means +/- SD indicated. Mann-Whitney test: p=0.5224 (ALT), p=0.6482 (AST), p=0.6601 (ALP) and p=0.8911 (albumin). (D) Histological assessment of pancreas from 1-year-old males by H&E staining. Data shown are representative of 3/3 mutants and 0/3 *WT* mice who developed pancreatic insulitis. Panels on the right show area-enlarged view of boxed area shown at lower magnification on the left, scale bar= 200µm.

Supplementary Figure 2 Mass cytometric immune profiling of splenocytes in 9 week-old mice. (A) *Left:* Exemplar contour plots show CD3 vs CD19 expression gated on live single splenic cells from each strain. Two-way ANOVA with post-hoc t-test was used for all genotype comparisons dis-aggregated by sex. *Right:* summary dot plots show absolute numbers of B, T, and non-T, non-B cells by sex and genotype. B cells: p=0.06 (females) vs p=0.34 (males). T cells: p=0.72 (females) vs p=0.72 (males). Non-T, non-B cells: p=0.56 (females) vs p=0.41 (males). (B) *Left:* Exemplar contour plots show CD122 vs NK1.1 expression gated on non-T non-B cells from each strain. *Right:* summary dot plots show absolute numbers of NK cells by sex and genotype: p=0.79 (females) vs p=0.79 (males). (C) *Left:* Exemplar contour plots show Sirpα vs Ly6G expression gated on non-T/B/NK cells from each strain. *Right:* summary dot plots show absolute numbers of granulocytes by sex and genotype: p=0.53 (females) vs p=0.11 (males). (D) *Left:* Exemplar contour plots show SiglecF vs CD64 expression gated on Sirpα^+^ Ly6G- non-dendritic cells from each strain. *Right:* summary dot plots show absolute numbers of SiglecF- CD64+ macrophages by sex and genotype: *p<0.05 (females) vs *p>0.05 (males). (E) *Left:* Exemplar contour plots show CD44 vs CD122 expression gated on CD8^+^ TCRαβ^+^ cells from each strain. *Right:* summary dot plots show absolute numbers of MP CD44^+^ CD122^+^ CD8 T cells by sex and genotype: p>0.99 (females) vs p=0.85 (males). For all dot plots, mean +/- SD are presented.

Supplementary Figure 3 Flow cytometric immune profiling of splenocytes and mLN in 15 week-old mice. Two-way ANOVA with post-hoc t-test was used for all genotype comparisons dis-aggregated by sex. (A) *Left:* Exemplar contour plots show CD4 vs CD8a expression gated on live single splenic CD3^+^ TCRβ^+^ cells from each strain. *Right:* summary dot plots show ratios of CD4/CD8 cells by sex and genotype. *p=0.01 (females) vs *p=0.01 (males). (B) *Left:* Exemplar contour plots show CD25 vs Foxp3 expression gated on CD4^+^ TCRβ^+^ T cells from each strain. *Right:* summary dot plots show absolute numbers of Foxp3^+^ T regulatory cells (Treg) cells by sex and genotype: p=0.53 (females) vs p=0.53 (males). (C) Summary dot plots show absolute numbers of CD62L- Foxp3^-^ CD4 T cells, and ICOS^+^ CD44^+^ CD62L^-^ CD4 T cells, from mLN by sex and genotype. Non-naïve CD4 cells: p=0.11 (females) vs p=0.14 (males). ICOS^+^ MP CD4 cells: **p=0.005 (females) vs *p=0.04 (males). (D) *Left:* Summary dot plots show absolute numbers of splenic CD19^+^ B220^+^ B cells by sex and genotype: p=0.15 (females) vs p=0.67 (males). *Middle:* Exemplar contour plots show CD21 vs CD24 expression gated on B cells from each strain. *Right:* summary dot plots show absolute numbers of follicular (CD21^med^ CD24^lo^), T1 (CD24^hi^ CD21^lo^) and T2 plus marginal zone (CD21^hi^ CD24^hi^) B cells by sex and genotype. Follicular B cells: p=0.09 (females) vs p=0.4 (males). T1 B cells: p=0.41 (females) vs p=0.94 (males). T2^+^marginal zone B cells: p=0.56 (females) vs p=0.79 (males). (E) Summary dot plots show absolute numbers of class-switched (IgM^lo^ IgD^lo^) B cells, activated pre-germinal center (GC; CD95^+^ GL7^-^) and GC (CD95^+^ GL7^+^) B cells, from mesenteric LN by sex and genotype. IgM^lo^ IgD^lo^ B cells: **p=0.002 (females) vs p=0.02 (males). CD95^+^ GL7^-^ B cells: **p=0.003 (females) vs p=0.15 (males). CD95^+^ GL7^+^ B cells: **p=0.001 (females) vs p=0.1 (males). For all dot plots, mean +/- SD are presented.

Supplementary Figure 4 ScRNA-seq analysis of 15 week-old mouse splenocytes. (A) Pre-gating strategy for each subset is shown on aggregated *WT* cells. *Left: * Dot plots show CD19 vs IgD protein expression showing gates for B cells (CD19^+^IgD^+^) vs. non-B cells (CD19^-^IgD^-^). Overlayed heatmap display *Ighm* expression which guided gating. *Middle: *CD4 vs TCRβ protein expression gated on CD19^-^IgD^-^ non-B cells with overlayed heatmaps showing Trac expression which guided gating decisions for CD4 cells (TCRβ^+^ CD4^+^) and CD8 (TCRβ ^+^ CD4^-^) cells. *Right:* CD44 vs CD62L protein expression gated on CD4^+^ TCRβ ^+^ T cells. Gates used for CD62L^+^CD44^-^ (naïve) and CD44^+^ CD62L^-^ MP CD4 cells are shown with overlayed heatmap of *Sell* encoding CD62L. (B) *Left:* summary dot plots show ratios of CD4/CD8 cells by genotype. Unpaired Student’s t-test with Welch’s correction p=0.16. *Right:* summary dot plots show ratios of CD62L^+^CD44^-^ / CD44^+^ CD62L^-^ CD4 cells by genotype, unpaired Student’s t-test with Welch’s correction **p<0.01. For all dot plots, mean +/- SD are presented. (C) *Left:* IgD vs CD44 protein expression on B cells from each strain, overlayed with heatmap of *Stat1*. *Middle:* Volcano plots show DEG between mutant and *WT* B cells, plotted according to Log_2_ fold change [Log_2_FC (mutant/*WT*)], against the negative logarithmic value of the false discovery rate [-log(FDR)]. Genes up- and down-regulated in mutant are denoted in red and blue, respectively. *Right:* Tables show genes up- or down regulated in mutant B cells by 40% or more. Log_2_FC values are presented along with FDR-adjusted q-values <0.05. (D) *Left:* dot plots show CD4 vs TCRβ protein expression gated on CD19^-^IgD^-^ non-B cells for aggregated samples from each strain, with overlayed heatmaps showing *Cd8b1* expression. *Middle:* Volcano plots show DEG between mutant and *WT* CD8 T cells, plotted according to Log2FC (mutant*/WT*)] against -log(FDR). Genes up- and down-regulated in mutant are denoted in red and blue, respectively. *Right:* Tables show genes up- or down regulated in mutant CD8 T cells by 40% or more. Log_2_FC values are presented along with FDR-adjusted q-values <0.05.
