# Supplementary figures and images for: The human Stat1 gain-of-function T385M mutation causes expansion of activated T-follicular helper/T-helper 1-like CD4 T cells and sex-biased autoimmunity in specific pathogen-free mice
Source: Front Immunol. 2023 May 19;14:1183273. doi: 10.3389/fimmu.2023.1183273 (PMC10235531; doi:10.3389/fimmu.2023.1183273)

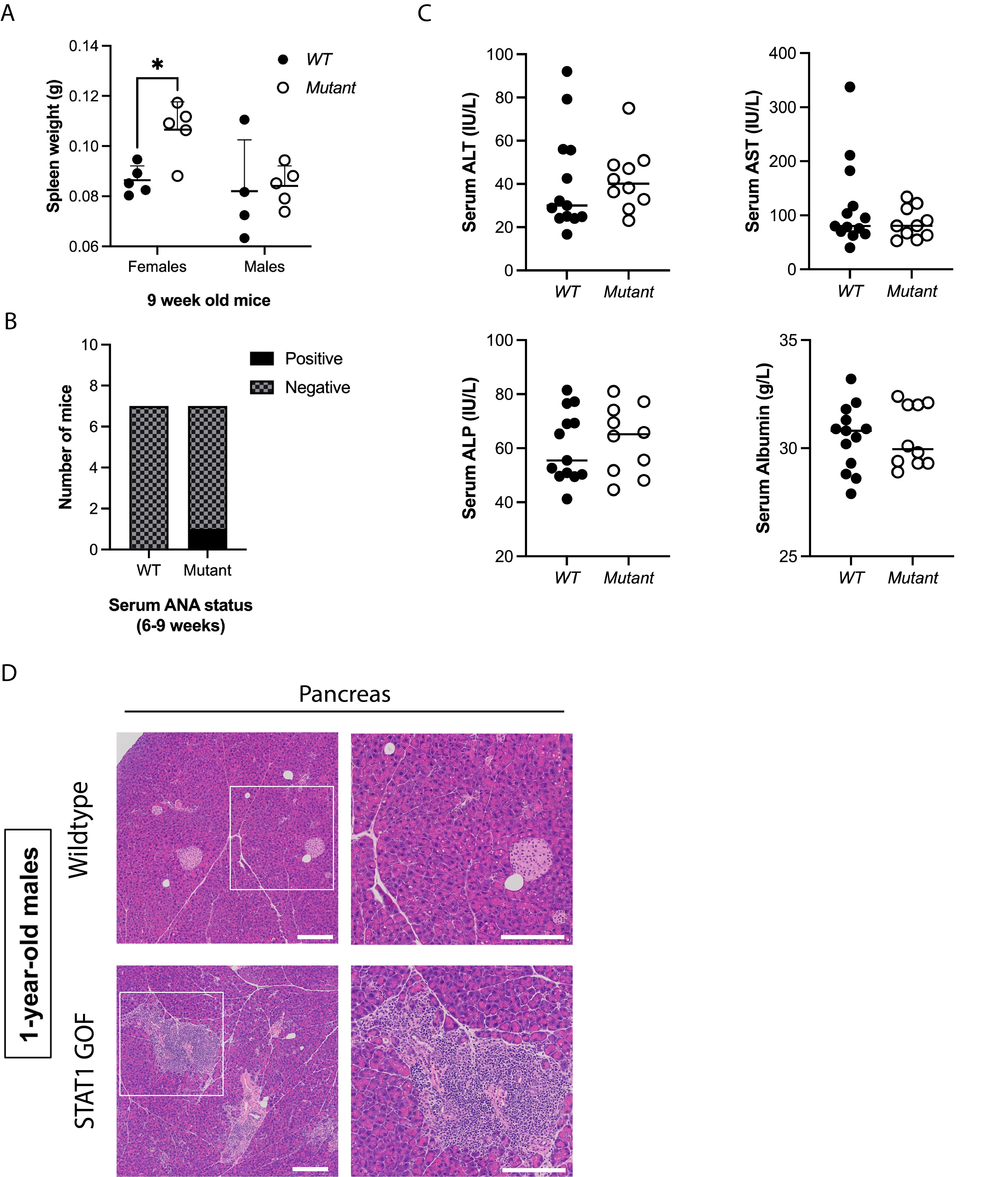

Supplement: Supplementary Figure 1 — Autoimmune manifestations in Stat1T385M/+ mice from 6 weeks to 1 year of age. (A) Dot plots show spleen weights in 9-week-old males and females, with means +/- SD indicated. Two-way ANOVA: FDR-adjusted p-values: *p<0.05 (females) vs p=0.42 (males). (B) Bar graphs show the observed frequency of serum positivity for anti-nuclear antibodies (ANA) as detected in 6-9-week-old mice via indirect immunofluorescence using HEp-2 substrate, at a screening dilution of 1:40. Fischer’s exact test **p>0.999. (C) Dot plots show serum values of liver parameters including alanine amino transferase (ALT), aspartate aminotransferase (AST), alkaline phosphatase (ALP) and albumin in 20 week-old mutants and WT littermates, with means +/- SD indicated. Mann-Whitney test: p=0.5224 (ALT), p=0.6482 (AST), p=0.6601 (ALP) and p=0.8911 (albumin). (D) Histological assessment of pancreas from 1-year-old males by H&E staining. Data shown are representative of 3/3 mutants and 0/3 WT mice who developed pancreatic insulitis. Panels on the right show area-enlarged view of boxed area shown at lower magnification on the left, scale bar= 200µm. [file Image_1.jpeg]

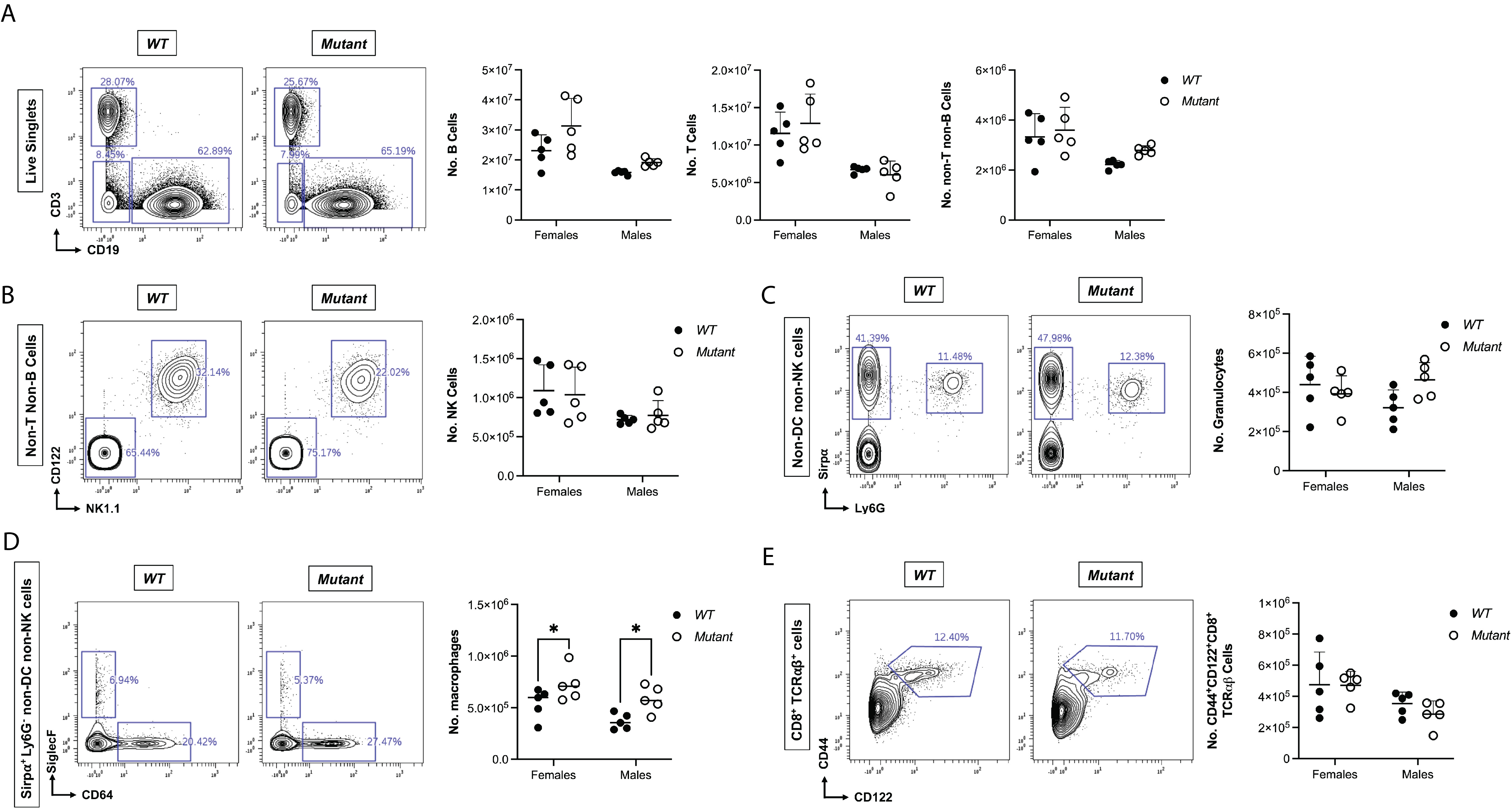

Supplement: Supplementary Figure 2 — Mass cytometric immune profiling of splenocytes in 9 week-old mice. (A) Left: Exemplar contour plots show CD3 vs CD19 expression gated on live single splenic cells from each strain. Two-way ANOVA with post-hoc t-test was used for all genotype comparisons dis-aggregated by sex. Right: summary dot plots show absolute numbers of B, T, and non-T, non-B cells by sex and genotype. B cells: p=0.06 (females) vs p=0.34 (males). T cells: p=0.72 (females) vs p=0.72 (males). Non-T, non-B cells: p=0.56 (females) vs p=0.41 (males). (B) Left: Exemplar contour plots show CD122 vs NK1.1 expression gated on non-T non-B cells from each strain. Right: summary dot plots show absolute numbers of NK cells by sex and genotype: p=0.79 (females) vs p=0.79 (males). (C) Left: Exemplar contour plots show Sirpα vs Ly6G expression gated on non-T/B/NK cells from each strain. Right: summary dot plots show absolute numbers of granulocytes by sex and genotype: p=0.53 (females) vs p=0.11 (males). (D) Left: Exemplar contour plots show SiglecF vs CD64 expression gated on Sirpα+ Ly6G- non-dendritic cells from each strain. Right: summary dot plots show absolute numbers of SiglecF- CD64+ macrophages by sex and genotype: *p<0.05 (females) vs *p>0.05 (males). (E) Left: Exemplar contour plots show CD44 vs CD122 expression gated on CD8+ TCRαβ+ cells from each strain. Right: summary dot plots show absolute numbers of MP CD44+ CD122+ CD8 T cells by sex and genotype: p>0.99 (females) vs p=0.85 (males). For all dot plots, mean +/- SD are presented. [file Image_2.jpeg]

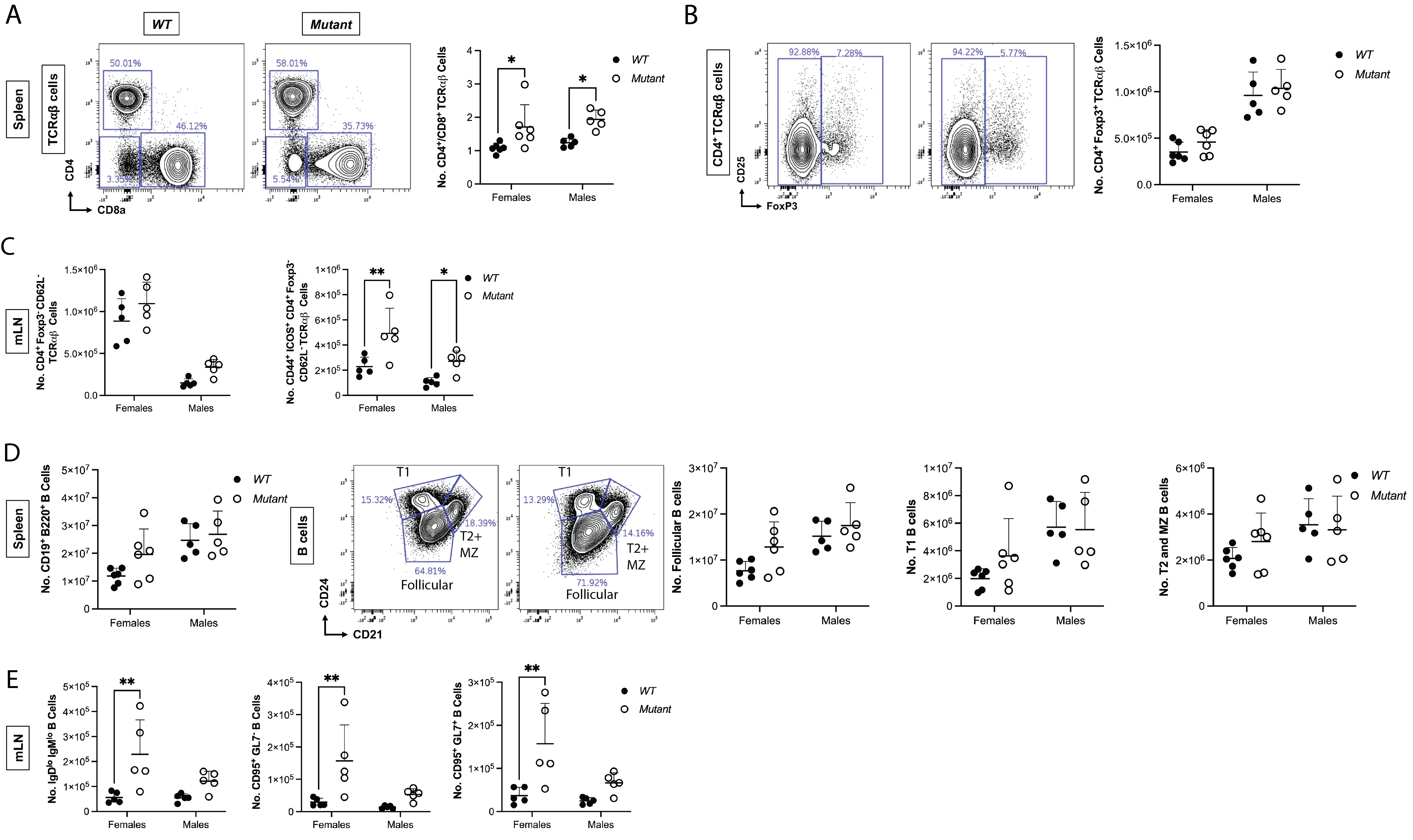

Supplement: Supplementary Figure 3 — Flow cytometric immune profiling of splenocytes and mLN in 15 week-old mice. Two-way ANOVA with post-hoc t-test was used for all genotype comparisons dis-aggregated by sex. (A) Left: Exemplar contour plots show CD4 vs CD8a expression gated on live single splenic CD3+ TCRβ+ cells from each strain. Right: summary dot plots show ratios of CD4/CD8 cells by sex and genotype. *p=0.01 (females) vs *p=0.01 (males). (B) Left: Exemplar contour plots show CD25 vs Foxp3 expression gated on CD4+ TCRβ+ T cells from each strain. Right: summary dot plots show absolute numbers of Foxp3+ T regulatory cells (Treg) cells by sex and genotype: p=0.53 (females) vs p=0.53 (males). (C) Summary dot plots show absolute numbers of CD62L- Foxp3- CD4 T cells, and ICOS+ CD44+ CD62L- CD4 T cells, from mLN by sex and genotype. Non-naïve CD4 cells: p=0.11 (females) vs p=0.14 (males). ICOS+ MP CD4 cells: **p=0.005 (females) vs *p=0.04 (males). (D) Left: Summary dot plots show absolute numbers of splenic CD19+ B220+ B cells by sex and genotype: p=0.15 (females) vs p=0.67 (males). Middle: Exemplar contour plots show CD21 vs CD24 expression gated on B cells from each strain. Right: summary dot plots show absolute numbers of follicular (CD21med CD24lo), T1 (CD24hi CD21lo) and T2 plus marginal zone (CD21hi CD24hi) B cells by sex and genotype. Follicular B cells: p=0.09 (females) vs p=0.4 (males). T1 B cells: p=0.41 (females) vs p=0.94 (males). T2+marginal zone B cells: p=0.56 (females) vs p=0.79 (males). (E) Summary dot plots show absolute numbers of class-switched (IgMlo IgDlo) B cells, activated pre-germinal center (GC; CD95+ GL7-) and GC (CD95+ GL7+) B cells, from mesenteric LN by sex and genotype. IgMlo IgDlo B cells: **p=0.002 (females) vs p=0.02 (males). CD95+ GL7- B cells: **p=0.003 (females) vs p=0.15 (males). CD95+ GL7+ B cells: **p=0.001 (females) vs p=0.1 (males). For all dot plots, mean +/- SD are presented. [file Image_3.jpeg]

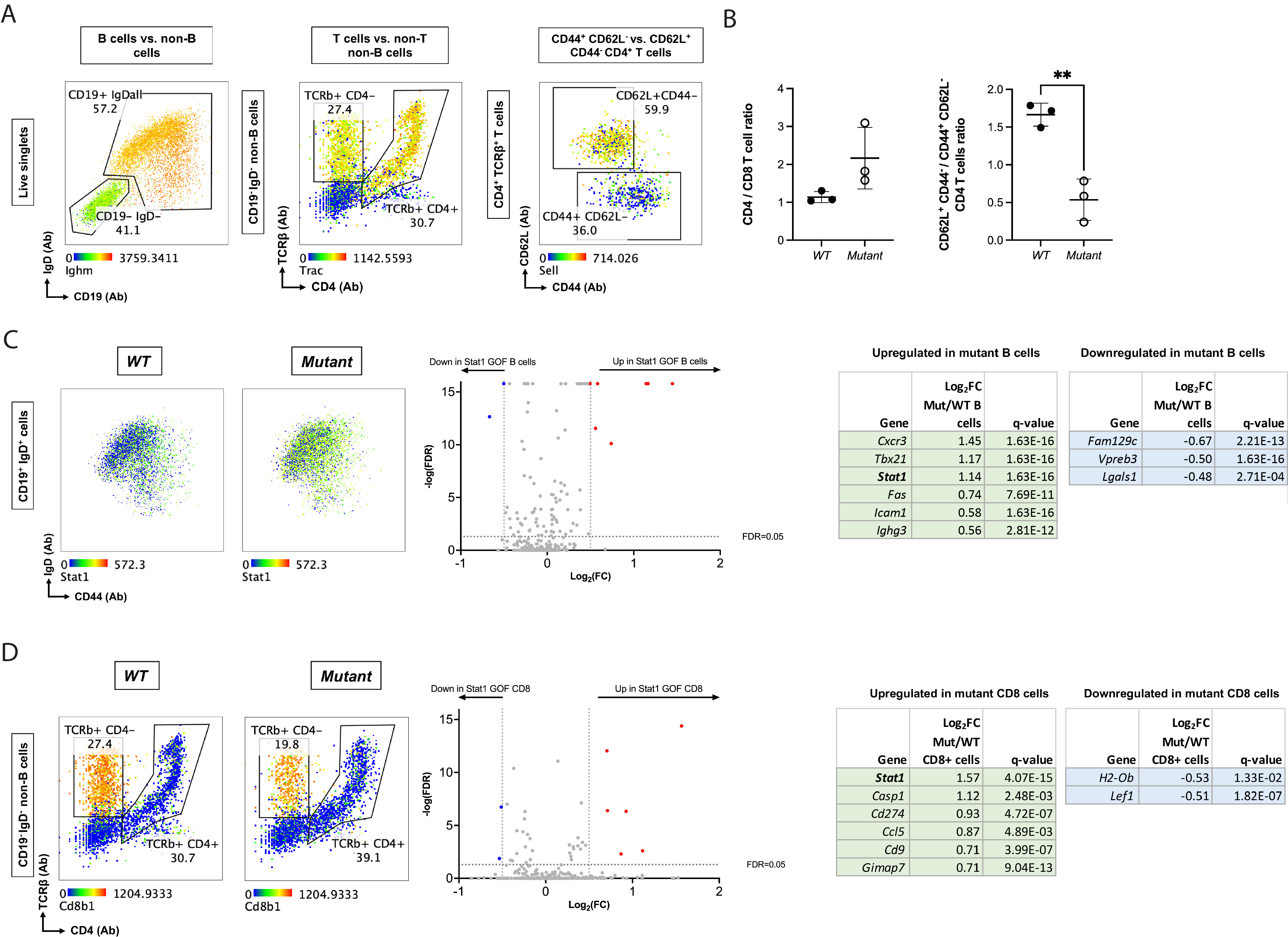

Supplement: Supplementary Figure 4 — ScRNA-seq analysis of 15 week-old mouse splenocytes. (A) Pre-gating strategy for each subset is shown on aggregated WT cells. Left: Dot plots show CD19 vs IgD protein expression showing gates for B cells (CD19+IgD+) vs. non-B cells (CD19-IgD-). Overlayed heatmap display Ighm expression which guided gating. Middle: CD4 vs TCRβ protein expression gated on CD19-IgD- non-B cells with overlayed heatmaps showing Trac expression which guided gating decisions for CD4 cells (TCRβ+ CD4+) and CD8 (TCRβ + CD4-) cells. Right: CD44 vs CD62L protein expression gated on CD4+ TCRβ + T cells. Gates used for CD62L+CD44- (naïve) and CD44+ CD62L- MP CD4 cells are shown with overlayed heatmap of Sell encoding CD62L. (B) Left: summary dot plots show ratios of CD4/CD8 cells by genotype. Unpaired Student’s t-test with Welch’s correction p=0.16. Right: summary dot plots show ratios of CD62L+CD44- / CD44+ CD62L- CD4 cells by genotype, unpaired Student’s t-test with Welch’s correction **p<0.01. For all dot plots, mean +/- SD are presented. (C) Left: IgD vs CD44 protein expression on B cells from each strain, overlayed with heatmap of Stat1. Middle: Volcano plots show DEG between mutant and WT B cells, plotted according to Log2 fold change [Log2FC (mutant/WT)], against the negative logarithmic value of the false discovery rate [-log(FDR)]. Genes up- and down-regulated in mutant are denoted in red and blue, respectively. Right: Tables show genes up- or down regulated in mutant B cells by 40% or more. Log2FC values are presented along with FDR-adjusted q-values <0.05. (D) Left: dot plots show CD4 vs TCRβ protein expression gated on CD19-IgD- non-B cells for aggregated samples from each strain, with overlayed heatmaps showing Cd8b1 expression. Middle: Volcano plots show DEG between mutant and WT CD8 T cells, plotted according to Log2FC (mutant/WT)] against -log(FDR). Genes up- and down-regulated in mutant are denoted in red and blue, respectively. Right: Tables show genes up- or down regulated in [file Image_4.jpeg]
